# Prehospital Factors Associated with Refractory Traumatic Arrest

**DOI:** 10.1155/2021/4624746

**Published:** 2021-12-20

**Authors:** Jeong Hun Lee, Yong Won Kim, Tae Youn Kim, Sanghun Lee, Han Ho Do, Jun Seok Seo, Seung Chul Lee

**Affiliations:** Department of Emergency Medicine, Dongguk University Ilsan Hospital, Dongguk University College of Medicine, Goyang, Republic of Korea

## Abstract

**Objective:**

Identification of the prehospital factors associated with a poor prognosis of immediate traumatic arrest should help reduce unwarranted treatment. We aim to reveal the clinical factors related to death after traumatic arrest on the scene.

**Methods:**

We performed a multicenter (4 tertiary hospitals in urban areas of South Korea) retrospective study on consecutive adult patients with trauma arrest on scene who were transferred by fire ambulance from January 2016 to December 2018. Patients with death on arrival in the emergency room (ER) were excluded. Prehospital data were collected from first aid records, and information on each patient's survival outcome in the ER was collected from an electronic database. Patients were divided into ER death and ER survival groups, and variables associated with prehospital trauma were compared.

**Results:**

A total of 145 (84.3%) and 27 (15.7%) patients were enrolled in the ER death and survival groups, respectively. Logistic regression analysis revealed that asystole (OR 4.033, 95% CI 1.342–12.115, *p* = 0.013) was related to ER death and that ROSC in the prehospital phase (OR 0.100, 95% CI 0.012–0.839, *p* = 0.034) was inversely related to ER death. In subgroup analysis of those who suffered fall injuries, greater height of fall was associated with ER death (15.0 (5.5–25.0) vs. 4.0 (2.0–7.5) meters, *p* = 0.001); the optimal height cutoff for prediction of ER death was 10 meters, with 66.1% sensitivity and 100% specificity.

**Conclusions:**

In cases of traumatic arrest, asystole, no prehospital ROSC, and falls from a greater height were associated with trauma death in the ER. Termination of resuscitation in traumatic arrest cases should be done on the basis of comprehensive clinical factors.

## 1. Introduction

Traumatic arrest immediately after injury is still usually irreversible and leads immediately to death despite the development of comprehensive modern trauma systems [1]. Immediate deaths account for about 60% of all trauma-related deaths. The overall survival rate from traumatic arrest on the prehospital phase is as low as 3.7%, and previous studies reported very poor outcomes even following cardiopulmonary resuscitation (CPR) [2]. Therefore, establishment of termination of resuscitation (TOR) rules for cases of immediate traumatic arrest may avoid unnecessary consumption of valuable resources and unwarranted treatment. The 2012 National Association of Emergency Medical Service (EMS) Physicians and American College of Surgeons' Committee on Trauma (NAEMSP-ASCOT) published a joint position article concerning TOR rules for patients in traumatic cardiac arrest [3]. However, these TOR rules cannot be applied uniformly across different countries because they consider not only the characteristics of traumatic arrest but also the traumatic care system in the prehospital phase. We aim to reveal the clinical factors related to death after traumatic arrest on the scene and validate the objective protocol of early TOR rules for traumatic arrest patients according to a variety of situations.

## 2. Methods

### 2.1. Characteristics of the Local EMS

In the local EMS, in which this study was conducted, primary care to major trauma victims on-site or during transfer is provided by three paramedics who are members of the fire department. This local EMS had protocols for traumatic arrest that included performing CPR on scene for 4 minutes and then transporting the patient to the nearest emergency room with ongoing CPR in the ambulance until ROCS. Invasive care (such as advanced airway management, external defibrillation, and intravenous fluid administration) by paramedics is supervised online by emergency medical physicians (EMPs) as needed. Because of the medical laws in Korea, paramedics cannot declare death even if supervised online by EMPs, so trauma resuscitation must be provided to traumatic arrest patients during the prehospital phase if the patients do not have injuries that are incompatible with life or obvious signs of death.

### 2.2. Study Population and Design

This is a retrospective observational multicenter study. This study enrolled consecutive patients with traumatic arrest who were over 18 years of age, had no response with apnea, who were pulseless on scene, and who were transferred by EMS to one of four tertiary hospital ERs located in Gyeonggi province of the Republic of Korea from January 2016 to December 2018. Patients were excluded if they did not undergo any resuscitation efforts in the emergency room (ER), if they were pronounced dead on arrival (DOA) because of confirmed injuries incompatible with life or obvious signs of death (such as livor mortis or rigor mortis), or if they had a do not resuscitate (DNR) order. Prehospital data were collected from the first aid records of the paramedics; this included injury information and information on the invasive care that was provided (age, sex, injury mechanism, witness, bystander CPR, EMS CPR, return of spontaneous circulation (ROSC), initial arrest electrocardiogram (ECG) rhythm, time factors of transportation, advanced airway, external defibrillation, and intravenous fluid). ER survival outcome was collected from the NEDIS (National Emergency Department Information System) electronic database. Shockable rhythm was defined as ventricular fibrillation or ventricular tachycardia on the initial arrest ECG rhythm. ER death was defined as death in the ER despite extensive resuscitation efforts. ER survival was defined as survival after the ER visit and subsequent management (surgical operation, transfer to a trauma center, or admission to an intensive care unit). We compared prehospital trauma-associated variables between the ER death and ER survival groups.

### 2.3. Statistical Analysis

Continuous variables are presented as median values (interquartile range) and compared using the Mann–Whitney test. Nominal data were calculated as percentages based on the frequency of occurrence and compared using the chi-square or Fisher's exact test, as appropriate. Multivariate logistic regression was performed to identify prehospital trauma-associated variables related to ER death in order to suggest criteria for early TOR rules. The resulting odds ratios (ORs) are presented with 95% confidence interval (95% CI). A two-sided *p* value of less than 0.05 is considered statistically significant. The statistical analyses were performed using IBM Statistical Package for the Social Sciences (SPSS) software version 24.0 (SPSS Inc., Chicago, IL, USA).

## 3. Results

**Figure 1:**
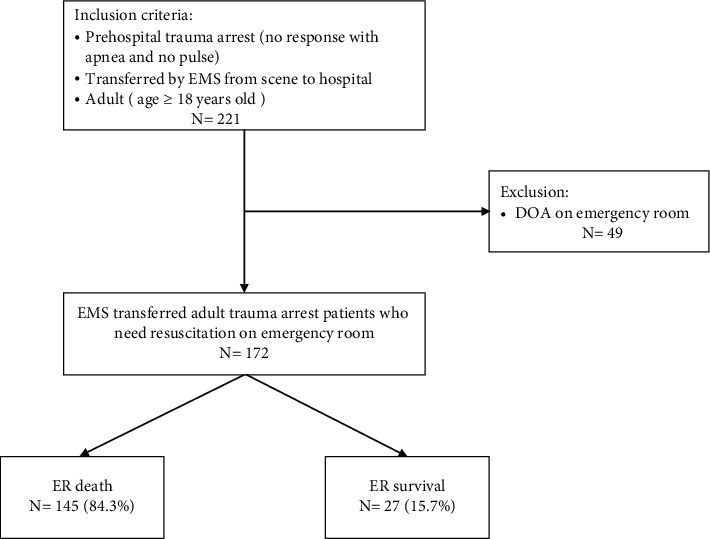
Flowchart of patient enrollment.

During the study period, 221 adult traumatic arrest victims were transferred by EMS from the incident scene to one of four tertiary hospital ERs. Of these 221 patients, 29 were excluded from the study because they were declared DOA at the ER. Finally, 172 patients were enrolled in the study (Figure 1).

After acute trauma care in the ER, 145 (84.3%) patients died in the ER, while the other 27 (15.7%) survived and received subsequent management for trauma. In the ER death group, the time from hospital arrival to death was median 72 (IQR 56–97) minutes. The baseline characteristics of the study subjects are given in Table 1.

Initial rhythm was different between the two groups. Asystole (59.3% vs. 33.3%) was more common in the ER death group than in the ER survival group, while PEA (51.9% vs. 34.5%) and shockable rhythm (7.4% vs. 3.4%) were more common in the ER survival group (*p* = 0.038). Regarding EMS management, defibrillation (14.8% vs. 3.4%) was more common in the ER survival group than in the ER death group. Prehospital ROSC (14.3% vs. 1.4%) was more common in the ER survival group.

Among the 68 patients with injuries related to falls, 59 (86.8%) belonged to the ER death group, and 9 (13.2%) were in the ER survival group. Height of the fall was greater in the ER death group (median 15.0, IQR 5.5–25.0 m vs. median 4.0, IQR 2.0–7.5 m, *p* = 0.001).

There were no significant differences in age, sex, injury mechanism, witness, bystander CPR, EMS CPR, advanced airway, intravenous fluid, and time factors of the prehospital phase between the two groups.

**Figure 2:**
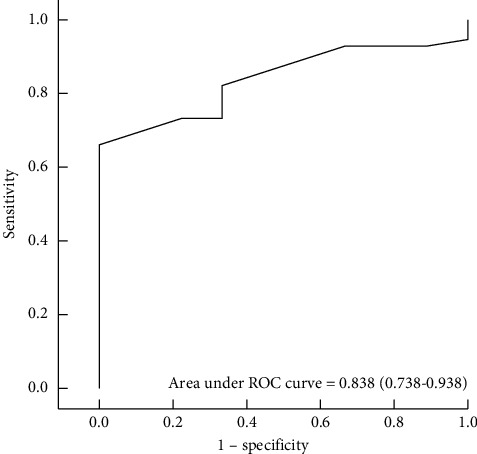
ROC curve for trauma death in the ER. The area under the curve for fall height was 0.838 (0.738–0.938). The optimal fall height cutoff value for prediction of trauma death in the ER was 10 meters with a sensitivity of 66.1% and a specificity of 100%. ROC, receiver operating characteristic; ER, emergency room.

The area under the receiver operating characteristic (ROC) curve describing the sensitivity and specificity of fall height for different cutoff levels was 0.838 (0.738–0.938) (Figure 2). The optimum height cutoff for prediction of trauma death at the emergency room was identified as 10 m, with a sensitivity of 66.1% and a specificity of 100%.

Multivariate analysis revealed that asystole (OR 4.033; 95% CI 1.342–12.115, *p* = 0.013) was related to trauma death in the ER and that ROSC in the prehospital phase (OR 0.100; 95% CI 0.012–0.839, *p* = 0.034) was inversely related to trauma death in the ER (Table 2).

When the early TOR rules consisted of asystole and no ROSC in the prehospital phase, the sensitivity was 58.6% (95% CI 50.2–66.7%), specificity was 70.37% (49.8–86.3%), positive predictive value was 91.4% (85.4–95.1%), and negative predictive value was 24.1% (18.2–30.2%) for trauma death in the ER.

## 4. Discussion

The TOR of traumatic arrest should be comprehensively determined by EMS providers or clinicians who take into consideration ethical issues, injury characteristics, condition of the patient, the local EMS system, and the overall medical environment. If there is no established guide to TOR, the point of termination of resuscitation may vary in accordance with the physician's opinion, even in similar situations. The region included in this study, Gyeonggi province of the Republic of Korea, has an area of approximately 10,000 km^2^ and a population of about 13 million and contains two trauma centers and six tertiary hospitals. Most patients with major trauma are transferred to the trauma center, but those with traumatic arrest are transferred to the nearest ER according to local EMS guidelines. All four tertiary hospitals in this study are located in urban areas, so the EMS arrival time at the scene and the time it took to get to the hospital were relatively short, approximately 8 minutes and 15 minutes, respectively. Since death cannot be declared during the prehospital phase under the current medical laws, the EMS providers did not terminate resuscitation on-site or during transfer. Also, there were no gun shot injuries because it is illegal for ordinary people to carry guns in Korea, so there were fewer cases of penetrating injury than in other countries.

We found that asystole and no prehospital ROSC despite EMS CPR are the predictors of poor prognosis of traumatic arrest, and these factors may provide a suitable basis on which to determine early TOR on ER. Similarly, several previous studies have reported that asystole is a poor prognostic factor, and although the studies population of these studies (e.g., penetrating injury to thorax, military service-related trauma) differ from our study, the mortality rate of trauma arrest with asystole is to 87–100% [4–7]. According to NAEMSP-ACSCOT (3), asystole is identified as an important factor on which to determine whether to withhold resuscitation in both penetrating and blunt trauma, and no ROSC despite appropriate EMS treatment is proposed in the TOR protocol. Our findings further showed that traumatic arrest caused by a fall from a height of more than 10 meters may not respond to resuscitation efforts. In general, the greater the height from which a person falls, the more serious his or her injuries and the higher the mortality rate [8, 9]. Thus, fall height is considered in most trauma centers as part of the criteria for trauma team activation [10]. Also, the reported mortality rates of falls from ≥ 6 meters, ≥12 meters, and ≥18 meters are 22.7%, 50%, and 100%, respectively [11, 12]. However, until now, little has been published about the prognosis in subgroups of patients who suffer from traumatic arrest at the scene due to a fall. Our findings showed that resuscitation efforts may be withheld or terminated if immediate traumatic arrest is caused by a fall from greater than 10 meters. Further studies may be needed that identify the situations in which resuscitation is futile in cases of traumatic arrest caused by other injury mechanisms.

Several studies have found that increases in EMS transfer time or in the duration of unsuccessful CPR are correlated with mortality in traumatic arrest, but it is difficult to suggest optimal cutoff time values for uniform TOR rules due to the heterogeneity of the study population [13–15]. Recently, the NAEMSP-ACSCOT suggested that up to 15 minutes of CPR should be provided before resuscitation efforts are terminated, but the evidence behind this suggestion remains unclear, and so they also suggested that further research is needed [3]. In our study, the median time from the scene to the hospital was 19.0 minutes (15.0–26.0) in the ER survival group; therefore, it may be inappropriate to terminate resuscitation efforts due to unsuccessful CPR even if the CPR time exceeds 15 minutes. Although not presented in our study, functional outcomes should also be considered when adding a time factor to the TOR rules because some past studies reported that longer CPR times were associated with poor neurological outcomes in cases of traumatic arrest even if the patient survived [16]. Factors that could prolong transport or CPR time include EMS management or hospital selection. Although the availability of EMS services generally improves the prognosis of major trauma, the survival outcomes following the provision of advanced life support by EMS providers remain controversial [17, 18]. In our study, there was no significant difference between invasive trauma surveys and outcome when treatment was given by EMS personnel. There was some evidence that transfer to the trauma center results in a better prognosis in the case of major trauma and that a reduction in EMS transport time is a very important prognostic factor in cases of traumatic arrest [19]. Further studies are required to identify the risks and benefits of choosing between an acceptable transport time and an ideal hospital (trauma center vs. nearest nontrauma facility) in traumatic arrest patients.

The present study had some limitations. First, as this was a retrospective review study, because of a lack of information about the data sources, we could not quantify either injury severity using an injury severity score (ISS) or the amount of in-hospital care that was provided, such as an emergency department thoracotomy. However, even if a prospective study is conducted, for patients who die in the emergency room due to traumatic arrest, an accurate ISS identification is difficult. Second, this study could not present long-term outcomes such as survival discharge and neurologic status. This is because the four institutions included in the study were not trauma centers, and the patients were often subjected to interfacility transfer for final treatment and rehabilitation.

## 5. Conclusion

In a trauma arrest patient on the scene, asystole, no prehospital ROSC, and fall from a greater height were associated with trauma death in the ER. The median transport time of ER survivors after traumatic arrest was 19 min, which was longer than the 15 min, currently considered to be nonsalvageable. In urban areas of South Korea, if asystole and no ROSC in the prehospital phase are applied as early TOR rules, ER mortality could be predicted with a specificity of 70.37% and a positive predictive value of 91.4%. In traumatic arrest, the TOR should be accompanied by decisions that take into account comprehensive clinical factors.

## Figures and Tables

**Table 1:** Baseline characteristics in the ER death and ER survival groups.

Parameters	Total, *N* = 172	ER death, *N* = 145 (84.3%)	ER survival, *N* = 27 (15.7%)	*P* value
Age (years)	53 (41–61)*∗*	54 (39–63)*∗*	52 (49–56)*∗*	0.099
Male, *N* (%)	122 (70.9)	104 (71.7)	18 (66.7)	0.646
Injury mechanism				0.065
Blunt, *N* (%)	169 (98.3)	144 (99.3)	25 (92.6)	
Penetration, *N* (%)	3 (1.7)	1 (0.7)	2 (7.4)	
Detail injury mechanism				
Fall down, *N* (%)	68 (39.5)	59 (40.7)	9 (33.3)	
Pedestrian versus motor vehicle, *N* (%)	41 (23.8)	35 (24.1)	6 (22.2)	
Motor vehicle occupant, *N* (%)	29 (16.9)	22 (15.2)	7 (25.9)	
Motorcycle, *N* (%)	17 (9.9)	17 (11.7)	0	
Other blunt injury, *N* (%)	14 (8.1)	11 (7.6)	3 (11.1)	
Stab injury	3 (1.7)	1 (0.7)	2 (7.1)	
Height of fall down (meter)	12.0 (5.0–24.0)*∗* *N*=68	15.0 (5.5–25.0)*∗* *N*=59	4.0 (2.0–7.5)*∗* *N*=9	0.001
Witness	84 (48.8)	70 (48.3)	14 (51.9)	0.835
Bystander CPR	78 (45.3)	65 (44.8)	13 (48.1)	0.834
EMS CPR	163 (94.8)	137 (94.5)	26 (96.3)	1.000
Initial rhythm				0.038
Asystole	95 (55.2)	86 (59.3)	9 (33.3)	
PEA	64 (37.2)	50 (34.5)	14 (51.9)	
VT or VF	6 (3.5)	4 (2.8)	2 (7.4)	
Unknown	7 (4.1)	5 (3.4)	2 (7.4)	
Advanced airway by EMS	96 (55.8)	80 (55.2)	16 (59.3)	0.833
Shockable rhythm with defibrillation by EMS	9 (5.2)	5 (3.4)	4 (14.8)	0.035
IV line with fluid resuscitation by EMS	35 (20.3)	27 (18.6)	8 (29.6)	0.200
ROSC on prehospital phase	6 (3.5)	2 (1.4)	4 (14.3)	0.006
Time from call to scene by EMS (min)	8.0 (6.0–11.0)*∗*	8.0 (6.0–11.0)*∗*	8.0 (6.0–10.5)∗	0.920
Time from scene to hospital by EMS (min)	15.5 (12.5–19.0)*∗*	15.0 (12.5–19.0)*∗*	19.0 (15.0–26.0)*∗*	0.222
Time from ER arrival to death (min)		72 (56–97)*∗*		

^∗^Median (interquartile range). ER, emergency room; CPR, cardiopulmonary resuscitation; EMS, emergency medical service; PEA, pulseless electrical activity; VT, ventricular tachycardia; VF, ventricular fibrillation; IV, intravenous; ROSC, return of spontaneous circulation.

**Table 2:** Multivariate analysis of prehospital factors related to ER death from traumatic arrest.

Predictors of ER death	Odds ratio	95% CI	*P* value
Age (years)	0.976	0.950–1.003	0.085
Male gender	1.744	0.638–4.769	0.278
Witness	1.623	0.556–4.735	0.376
Bystander CPR	1.396	0.489–3.980	0.533
EMS CPR	0.284	0.023–3.482	0.325
Blunt injury	10.065	0.501–202.079	0.131
Asystole	4.033	1.342–12.115	0.013
Supraglottic airway	0.861	0.326–2.274	0.763
Need of defibrillation	0.345	0.061–1.943	0.228
Fluid resuscitation	0.487	0.159–1.488	0.207
ROSC on prehospital phase	0.100	0.012–0.839	0.034
Duration of transfer by EMS (min)	0.974	0.920–1.031	0.371

ER, emergency room; CI, confidence interval; CPR, cardiopulmonary resuscitation; EMS, emergency medical service; ROSC, return of spontaneous circulation.

## Data Availability

The data used to support the findings of this study are available from the corresponding author upon request.
